# Evolution of Rapid Clonal Dynamics and Non–Cross-Resistance in Response to Alternating Targeted Therapy and Chemotherapy in BRAF-V600E-Mutant Colon Cancer

**DOI:** 10.1200/PO.23.00260

**Published:** 2024-12-03

**Authors:** Srilatha Simhadri, Jillian N. Carrick, Susan Murphy, Om A. Kothari, Husam Al-Hraishami, Atul Kulkarni, Nahed Jalloul, Katarina Stefanik, Manisha Bandari, Kavya Chettur, Ming Yao, Vasudeva Ginjala, Roman Groisberg, Howard Hochster, Janice Mehnert, Gregory Riedlinger, Hossein Khiabanian, Michael P. Verzi, Kevin Tong, Shridar Ganesan

**Affiliations:** ^1^ Department of Medicine, Rutgers Cancer Institute of New Jersey, Robert Wood Johnson Medical School, Rutgers University, New Brunswick, NJ; ^2^ Department of Genetics, Human Genetics Institute of New Jersey, Rutgers University, Piscataway Township, NJ; ^3^ Hackensack Meridian Health Center for Discovery and Innovation, Nutley, NJ; ^4^ Department of Medicine, Pediatric Hematology/Oncology Program, Rutgers Cancer Institute of New Jersey, Robert Wood Johnson Medical School, Rutgers University, New Brunswick, NJ; ^5^ Department of Pathology, Rutgers Cancer Institute of New Jersey, Robert Wood Johnson Medical School, Rutgers University, New Brunswick, NJ; ^6^ Department of Biology, The College of New Jersey, Ewing, NJ; ^7^ Tenafly High School, Tenafly, NJ; ^8^ Department of Medical Sciences, Hackensack Meridian Health School of Medicine, Nutley, NJ

## Abstract

**PURPOSE:**

Combined BRAF, MEK, and EGFR inhibition can induce clinical responses in BRAF-V600E-mutant colon cancer, but rapid resistance often occurs.

**METHODS:**

We use serial monitoring of circulating tumor DNA cell-free plasma DNA (cfDNA) in a patient case study in addition to organoids derived from mouse models of BRAF-V600E-mutant intestinal cancer, which emulated the patient's mutational profile to assess drug treatment efficacy.

**RESULTS:**

We demonstrate dynamic evolution of resistance to combined EGFR/BRAF/MEK inhibition in a pediatric patient with metastatic BRAF-V600E-mutant, mismatch repair-stable colon cancer. Initial resistance to targeted therapy was associated with development of MET amplification. Sequential treatment with chemotherapy and targeted therapy resulted in clearing of the resistant MET-amplified clone. Rechallenge with combined BRAF/EGFR inhibition resulted in clinical and radiographic response, demonstrating these treatments may be non–cross-resistant. Tumor organoids were used to model clinical findings and demonstrated effectiveness of combined targeted therapy and chemotherapy.

**CONCLUSION:**

These findings suggest rational strategies for combining sequential chemotherapy and BRAF-/EGFR-directed therapy in BRAF-V600E-mutant colon cancer to prevent resistance and improve outcome. The data demonstrate rapid clonal dynamics in response to effective therapies in BRAF-V600E-mutant colon cancer that can be monitored by serial cfDNA analysis. Moreover, in mismatch repair-proficient BRAF-V600E-mutant colon cancers, combined EGFR and BRAF/MEK therapy is not cross-resistant with standard chemotherapy, suggesting new rational combination treatment strategies.

## Teaser

Rapid assessment of tumor clonal dynamics in cfDNA suggests altering current treatment strategies.

## INTRODUCTION

Approximately 8%-15% of colorectal cancers (CRC) harbor a BRAF-V600E mutation, which is associated with aggressive disease, poor prognosis, and does not show significant response to standard therapy nor BRAF/MEK inhibitors.^1-7^ A subset of metastatic BRAF-V600E-mutant colon cancers also show evidence of microsatellite instability and defective mismatch repair because of *hMLH1* promoter hypermethylation and the CpG island methylator phenotype.^1,2^ Likely because of adaptive activation of EGFR signaling in the setting of mitogen-activated protein kinase pathway inhibition, BRAF-V600E-mutant colon cancers generally do not benefit from addition of anti-EGFR antibody to standard chemotherapy.^1,2^ However, a phase III trial demonstrating that a doublet of BRAF inhibitor with an EGFR antibody, and triplet of BRAF, MEK, and EGFR inhibitors, resulted in significantly longer overall survival and higher response rate than standard chemotherapy in patients with BRAF-V600E metastatic colorectal cancer.^8^ However, these responses are often short-lived and acquired resistance develops through multiple mechanisms.^9^ Thus, there is a pressing need to develop novel strategies to improve the durability of treatment responses and target resistance mechanisms.

Here, we report a case of primary BRAF-V600E-mutant, microsatellite-stable (MSS), metastatic CRC in a 16-year-old female patient who was treated with alternating chemotherapy and EGFR-/BRAF-/MEK-targeted therapy on the basis of evidence of tumor evolution monitored by serial cell-free plasma DNA (cfDNA) sequencing assays. These treatments are recapitulated using organoids from BRAF-V600E-mutant mouse models.^10^ Our findings suggest that chemotherapy and targeted therapy are non–cross-resistant and induce quite distinct selection pressures in BRAF-V600E-mutant CRC. We demonstrate how alternating therapies may lead to rapid changes in clonal dynamics that can be monitored by cfDNA assays and may lead to new rationally designed treatment strategies.

**CONTEXT**

**Key Objective**
How does clonal dynamics in solid tumors affect drug resistance and sensitivity during treatment regimens?
**Knowledge Generated**
Different clonal populations can dominate and recede in BRAF-mutant colon cancer because of selection pressure from targeted therapy and chemotherapy and can be monitored by serial cfDNA assays. Targeted therapy with BRAF/MEK/EGFR inhibition may be non–cross-resistant to standard chemotherapy in BRAF-mutant colon cancer, and monitoring of clonal dynamics during therapy may allow rational rechallenge strategies.
**Relevance**
Serial circulating tumor DNA analysis can demonstrate rapid clonal dynamics that evolve during response and resistance to both standard chemotherapy and targeted therapy of BRAF-mutant colon cancer and can suggest new rational treatment strategies.

## METHODS

### Patient Case Study

The patient was enrolled in a consented, prospective clinical sequencing protocol at the Rutgers Cancer Institute of New Jersey (ClinicalTrials.gov identifier: NCT02688517). Tumor sequencing was done using the commercial FoundationOne Assay, a hybrid capture–based tumor sequencing panel. Serial plasma cfDNA assays were performed using the Guardant 360 Assay. Tumor measurements were performed on radiographic images using measurement tools in DICOM. The protocol was approved by the Institutional Review Board of Rutgers Robert Wood Johnson Medical School.

### Animals and Organoid Cultures

Animal experiments were conducted in accordance with Rutgers University IACUC. Smad4^f/f^; Braf^V600E/+^; *Villin-Cre*^*ERT2*^ mice^10-12^ were treated with intraperitoneal injection of tamoxifen (1 mg/20 g, once daily) for four consecutive days. Organoids were derived from uninjected control mice duodenal crypts and cultured in Cultrex reduced growth factor matrix R1 (BME-R1, Trevigen, Minneapolis, MN) and 1× Crypt Culture Medium (CCM) as previously described.^10-13^ Tumor organoids were derived from Smad4^f/f^; Braf^V600E/+^ mice as previously described.^11,12,14^

### Organoid Treatments

Organoids were passaged after 7 days using TrypLE (Gibco, Waltham, MA). Approximately 100 organoids per biologic replicate were seeded and cultured in 1× CCM for 3 days. Viable organoids were counted, and organoids were treated with 1× CCM supplemented with vehicle (0.03% DMSO), vemurafenib (PLX4032, SelleckChem, Houston, TX), erlotinib (SelleckChem), FU (fluorouracil; SelleckChem), SN-38 (SelleckChem), crizotinib (SelleckChem), or combinations of inhibitors for 7-14 days, exchanging media every 2 days. Images were taken using light microscopy with iPhone rear-facing cameras.

### Generation of MET-Expressing Tumor Organoids

Lentiviral transduction of tumor organoids was performed using established protocol^15^ with modifications. Lentiviral packaging vectors (pVSVg, Δ8.9), mock or pLenti-MetGFP plasmid (gift from David Rimm, Addgene plasmid #37560), and Lipofectamine2000 (ThermoFisher, Waltham, MA) were incubated with HEK293T cells per manufacturer protocol for 16 hours. Cells were then incubated for 72 hours, collecting and replacing media after 48 hours for virus collections. Passaged organoids were incubated with high-titer virus in 1× CCM plus 10 mM nicotinamide, 10 µM Chir99021, 10 µM Y27632, and 8 μg/mL polybrene for 4 hours. Transduced organoids were seeded in BME-R1 and cultured in 1× CCM, 10 mM nicotinamide, 10 µM Chir99021 and 10 µM Y27632, and 2 μg/mL Puromycin (Gibco) for selection. Quantitative polymerase chain reaction validation for cMET was performed. Primers: MET_F: 5′-AAG​AGG​GCA​TTT​TGG​TTG​TG-3′; MET_R: 5′-GAT​GAT​TCC​CTC​GGT​CAG​AA-3′.

### Ethics Approval and Consent to Participate

Patient was enrolled in a consented, prospective clinical sequencing protocol at the Rutgers Cancer Institute of New Jersey (ClinicalTrials.gov identifier: NCT02688517).

### Consent for Publication

All authors have reviewed and confirmed their consent for publication.

## RESULTS

### Response and Acquired Resistance to Combined EGFR-, BRAF-, and MEK-Targeted Therapy in BRAF-V600E-Mutant CRC

**FIG 1.**
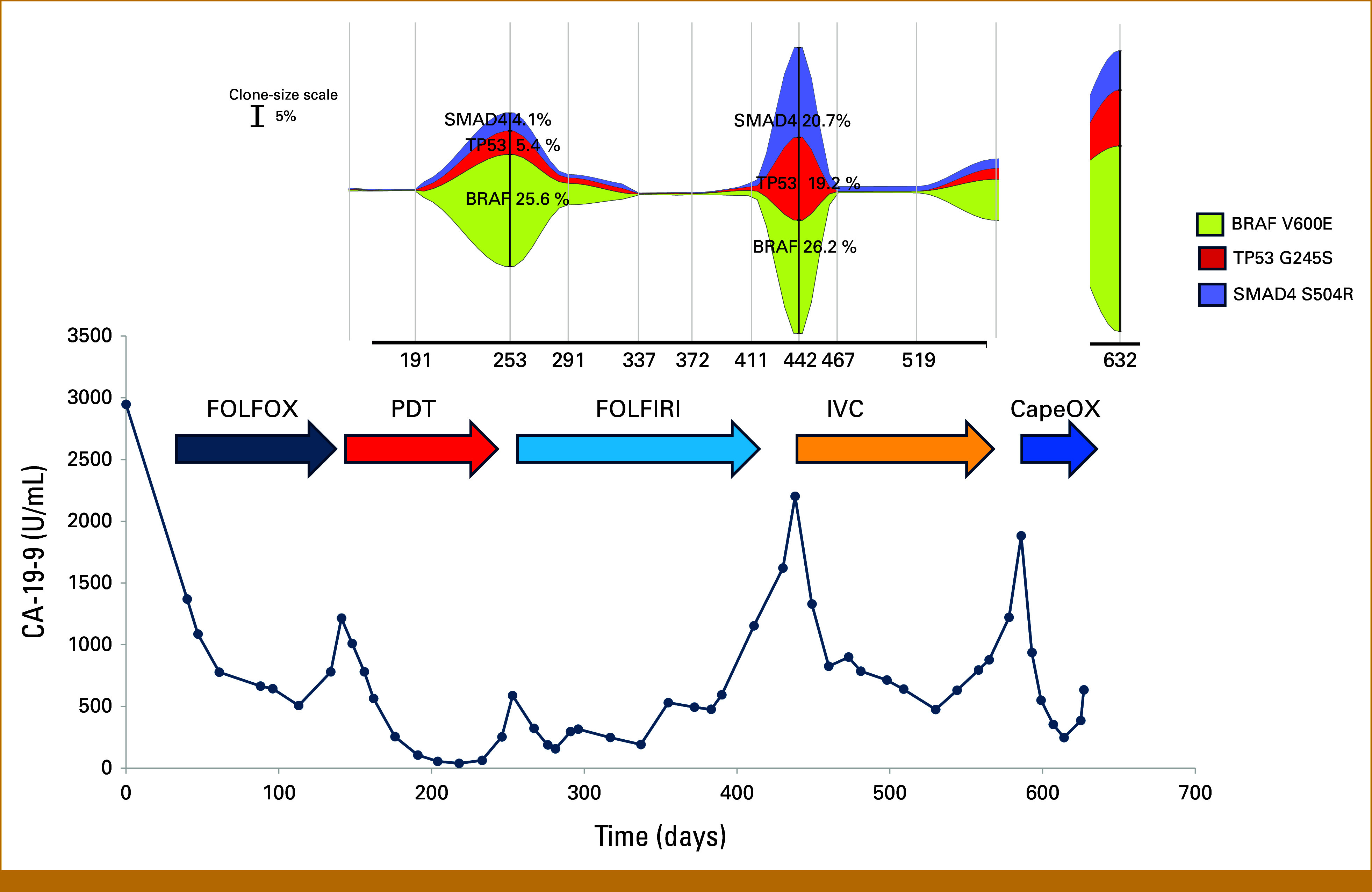
Response and resistance to serial therapeutic regimens in a patient with BRAF-V600E-mutant colon cancer, and CA19-9 levels measured over the course of treatment are plotted. Onset and time of different treatment regimens used are shown by colored arrows. In the inset on top is shown a plot of the relative allele frequencies of specific mutations identified in the plasma cfDNA assay on the same time scale. CapeOX, capecitabine, oxaliplatin; cfDNA, cell-free DNA; FOLFIRI, leucovorin, fluorouracil, irinotecan; FOLFOX, leucovorin, fluorouracil, oxaliplatin; IVC, irinotecan, vemurafenib, cetuximab; PDT, panitumumab, dabrafenib, trametinib.

**FIG 2.**
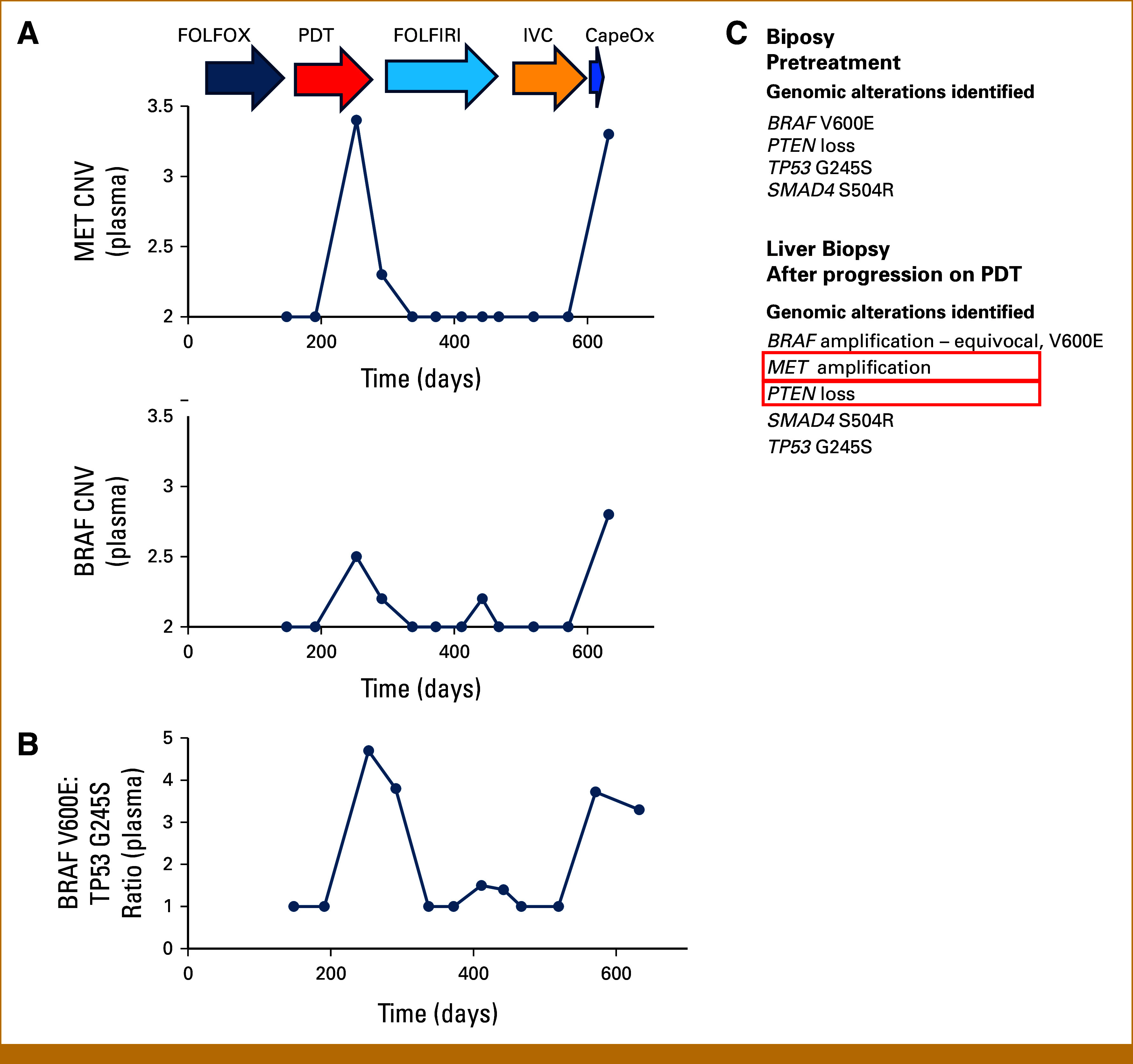
Evolution of genomic amplifications in response to therapy. (A) Calculated copy numbers of MET and BRAF in plasma cfDNA samples are shown at indicated timepoints in course of treatment. (B) Ratio of the MAF of BRAF-V600E and TP53 G245S is plotted over time. (C) Sequencing results from FoundationOne tumor sequencing panels from the hepatic flexure biopsy specimen obtained before initial treatment and liver biopsy after development of resistance to PDT therapy. CapeOX, capecitabine, oxaliplatin; CNV, copy number variant; cfDNA, cell-free DNA; FOLFIRI, leucovorin, fluorouracil, irinotecan; FOLFOX, leucovorin, fluorouracil, oxaliplatin; IVC, irinotecan, vemurafenib, cetuximab; PDT, panitumumab, dabrafenib, trametinib.

A 16-year-old girl (no family history nor history of childhood cancers) presented in 2015 with recurrent abdominal pain, jaundice, and signs of intestinal obstruction. A mass was found in the ascending colon as well as multiple liver lesions with associated biliary obstruction, concerning for metastatic colon cancer. She underwent biliary stent placement by endoscopic retrograde cholangiopancreatography. Biopsies of liver and colon lesions showed moderately differentiated colon cancer; immunohistochemistry analysis showed intact expression of usual mismatch repair proteins (data not shown). She was started on infusional fluorouracil, leucovorin, and oxaliplatin (FOLFOX) therapy, with the course complicated early by impending bowel obstruction requiring an emergency diverting ileostomy; she continued on FOLFOX and appeared to respond as observed by the reduction in the levels of the biomarker, serum cancer antigen CA19-9 (Fig 1). However, in 3 months, she developed evidence of progression as seen by rising CA19-9 levels (Fig 1). The patient was enrolled on a tumor sequencing protocol (ClinicalTrials.gov identifier: NCT02688517), and comprehensive genomic profiling analysis revealed the presence of BRAF-V600E mutation, TP53-G245S, SMAD4-S504R, and PTEN loss (Fig 2). The Rutgers Cancer Institute Molecular Tumor Board (RCIMTB) reviewed this case in late 2015, and recommended treatment with a combination of panitumumab, dabrafenib, and trametinib (PDT) on the basis of the absence of an available clinical trial or pediatric study results, known poor response to second-line chemotherapy, and data from early-phase trials in adults at the time.^16^

**FIG 3.**
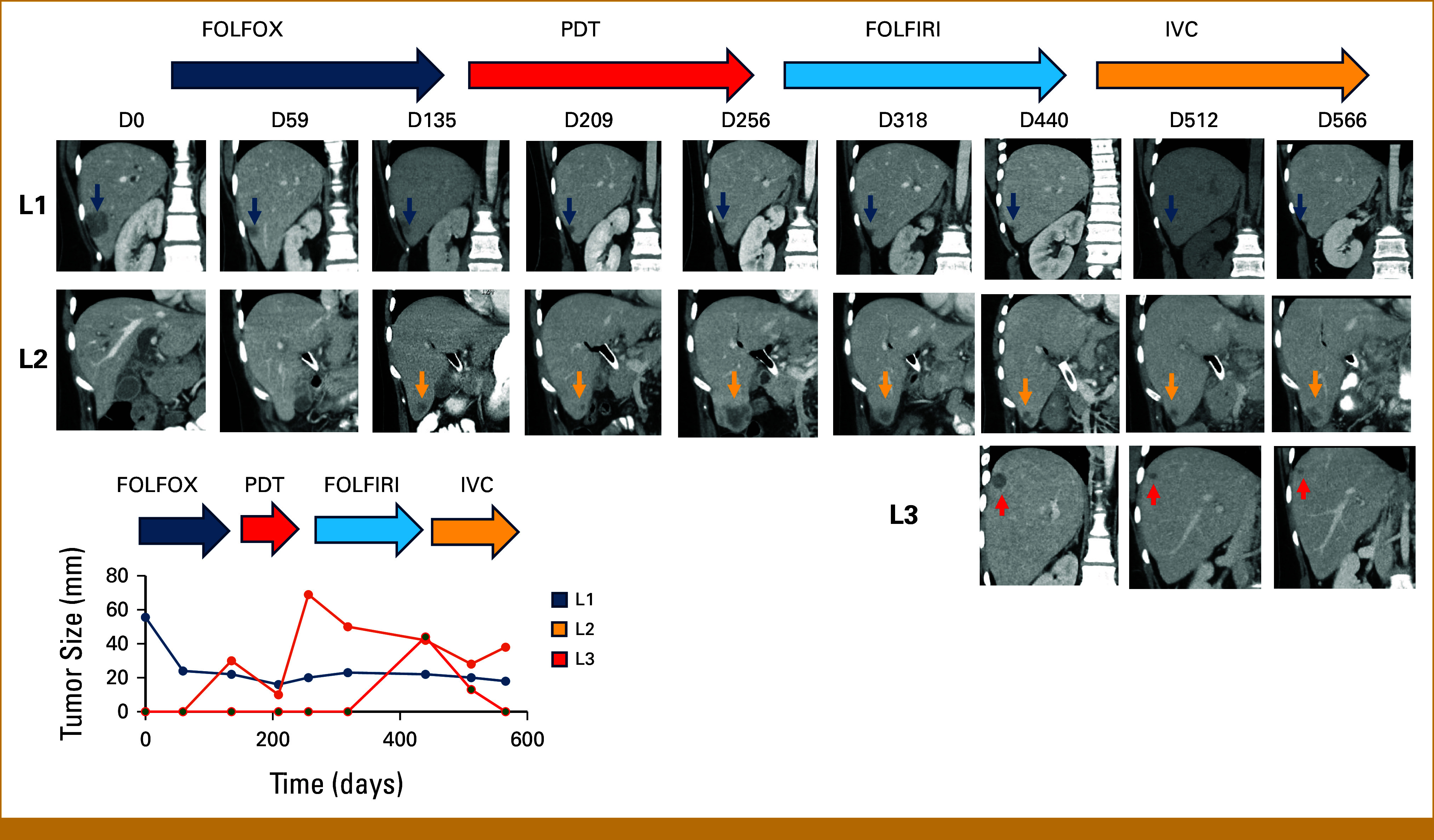
Spatial heterogeneity of response and resistance. Serial radiographs highlighting the history of three separate liver metastases over time, with treatments and time of images indicated. Inset graph shows measurement of tumor size, as measured by the sum of two orthogonal linear measurements for each lesion in mm, over time. CapeOX, capecitabine, oxaliplatin; FOLFIRI, leucovorin, fluorouracil, irinotecan; FOLFOX, leucovorin, fluorouracil, oxaliplatin; IVC, irinotecan, vemurafenib, cetuximab; PDT, panitumumab, dabrafenib, trametinib.

The patient responded to the PDT regimen, with rapid reduction in CA19-9 levels and reduction in liver metastases (Figs 1 and 3), but developed grade 3 acneiform rash, leading to dose reduction of panitumumab. Concurrent serial monitoring of cfDNA, starting at D148, showed low mutant allele frequencies of BRAF-V600E (0.2%) at the initial response to PDT, with BRAF-V600E being undetectable at the CA19-9 nadir on D191 (Fig 1, Appendix Table A1). After approximately 3 months of PDT therapy, there was evidence of disease progression with rising CA19-9 levels and development of new liver lesions (Figs 1 and 3). cfDNA assays and sequencing of new liver lesion biopsy showed increase in BRAF-V600E mutant allele frequency, with evidence of BRAF and MET amplification (Fig 2), both known mechanisms of acquired resistance to PDT therapy.^9,17^ There was persistent absence of microsatellite instability (MSI). The patient was again reviewed at the RCIMTB, and crizotinib treatment was considered, having shown activity in this setting.^17^ However, since the patient rapidly progressed before crizotinib therapy could be initiated, she was started on standard second-line chemotherapy with a FU and irinotecan-based regimen (FOLFIRI).

On FOLFIRI therapy, the patient initially responded as seen by declining CA19-9 levels as well as a drop in cfDNA BRAF-V600E mutant allele frequencies (0.1%, Fig 1, Appendix Table A1). However, after 3 months on FOLFIRI, she again had rising CA19-9 and increasing BRAF-V600E mutant allele frequencies (26.2%), consistent with progression and acquired resistance to FOLFIRI. Despite rising allele frequencies of BRAF-V600E, there was no evidence of BRAF or MET amplification (Fig 2). This suggested that the dominant clones, although now resistant to FOLFIRI, would no longer be resistant to therapy targeting the BRAF-V600E mutation. As some lesions that responded to FOLFIRI remained controlled, and progression was associated with development of new lesions (Fig 3), it was decided to use a combination of irinotecan, vemurafenib, and cetuximab (IVC), a regimen that continued irinotecan and added BRAF- and EGFR-targeted therapy, which was shown to be safe and efficacious in phase IB trials.^18^

The patient's cancer responded to rechallenge with BRAF- and EGFR-targeted therapy with a rapid decline in CA19-9 levels, BRAF-V600E allele frequencies (0.3%-0.4%), as well as a radiographic response of the new lesions (Figs 1 and 3). However, after about 3 months of therapy, CA19-9 levels rose with concomitant increase in BRAF-V600E allele frequencies (9.3%), and ultimately evidence of disease progression (Figs 1 and 3, Appendix Table A1). Although relative BRAF-V600E allele frequencies were increased compared with preresistance values, suggesting BRAF amplification, no evidence of MET amplification was seen. Given the previous excellent response to second-line chemotherapy, the patient was re-treated with capecitabine and oxaliplatin. Although initial response was seen, this lasted only several weeks and the patient rapidly had disease progression (Fig 1), and died shortly thereafter because of complications from tumor progression, including biliary obstruction and infection. cfDNA analysis at the time of final progression showed presence of both BRAF and MET amplification (Figs 1 and 2).

### Dynamics of cfDNA Mutant Allele Frequencies During Course of Therapy

Serial analyses of cfDNA performed every 2-3 months from D148 to D632 show that overall mutant allele frequencies (MAFs) of BRAF, TP53, and SMAD directly correlate with levels of the serum tumor marker CA19-9 and radiographic results (Figs 1 and 3). Responses to therapy were associated with decreases in MAF and progression associated with increasing MAFs. The lowest levels of CA19-9 were associated with similar nadirs in MAF. Of note, the ratio of BRAF-V600E MAF to TP53 G245S MAF was elevated in cfDNA results, consistent with amplification of the mutant BRAF-V600E allele at that time (Fig 2B).

Mutations in PIK3CA (E453D), ERBB2 (C584G), CCNE1 (A353D), ARID1A (R693R), AR (I665M), APC (R120I), NOTCH1 (E1636K), and NTRK (E581K) were each seen at only one time point and at very low allele frequencies (<1%, Appendix Table A1). As these were missense (or synonymous) mutations of unclear significance, and detected at only one time point, it is not clear if they represent true subclones or were false-positive calls.

Evidence of MET amplification receded after initiation of FOLFIRI and clinical response. When the tumor acquired resistance to FOLFIRI with radiographic evidence of tumor progression and rising MAFs of BRAF, TP53, and SMAD (Fig 1), there was no evidence of MET amplification (Fig 2A). Of note, there was some increase in BRAF copy number, although a skewed ratio of BRAF-V600E to TP53 G245S MAF was not seen. However, at final progression shortly after treatment with IVC, MET and BRAF amplifications, with skewed BRAF-V600E:TP53 ratio, were again seen.

### Spatial Heterogeneity of Metastatic Response

Individual metastatic lesions showed different patterns of response and recurrence, suggesting different clonal compositions (Fig 3). Some liver lesions (L1) that were present at diagnosis regressed in response to initial FOLFOX therapy and never regrew, while other lesions (L2) showed more complex dynamics. Lesion L2 developed during FOLFOX treatment at D135 initially responded to PDT as seen on imaging at D209, but showed progression at D256. This lesion then responded to FOLFIRI (D318 and D440), but ultimately progressed on IVC treatment (D566). This suggests that lesion L2 contained a mixture of clones that were differentially sensitive to both chemotherapy and EGFR/BRAF/MEK inhibition. Lesion L3, by contrast, developed late during FOLRIRI treatment (D440), and responded rapidly to the addition of BRAF and EGFR inhibition with IVC regimen (D512, D566). These data show that individual metastatic lesions are mixtures of sensitive and resistant clones, and that clones resistant to chemotherapy and targeted therapy are not homogeneously distributed at all metastatic sites.

### Organoid Model of BRAF-V600E-Mutant Cancer Recapitulates Resistances

**FIG 4.**
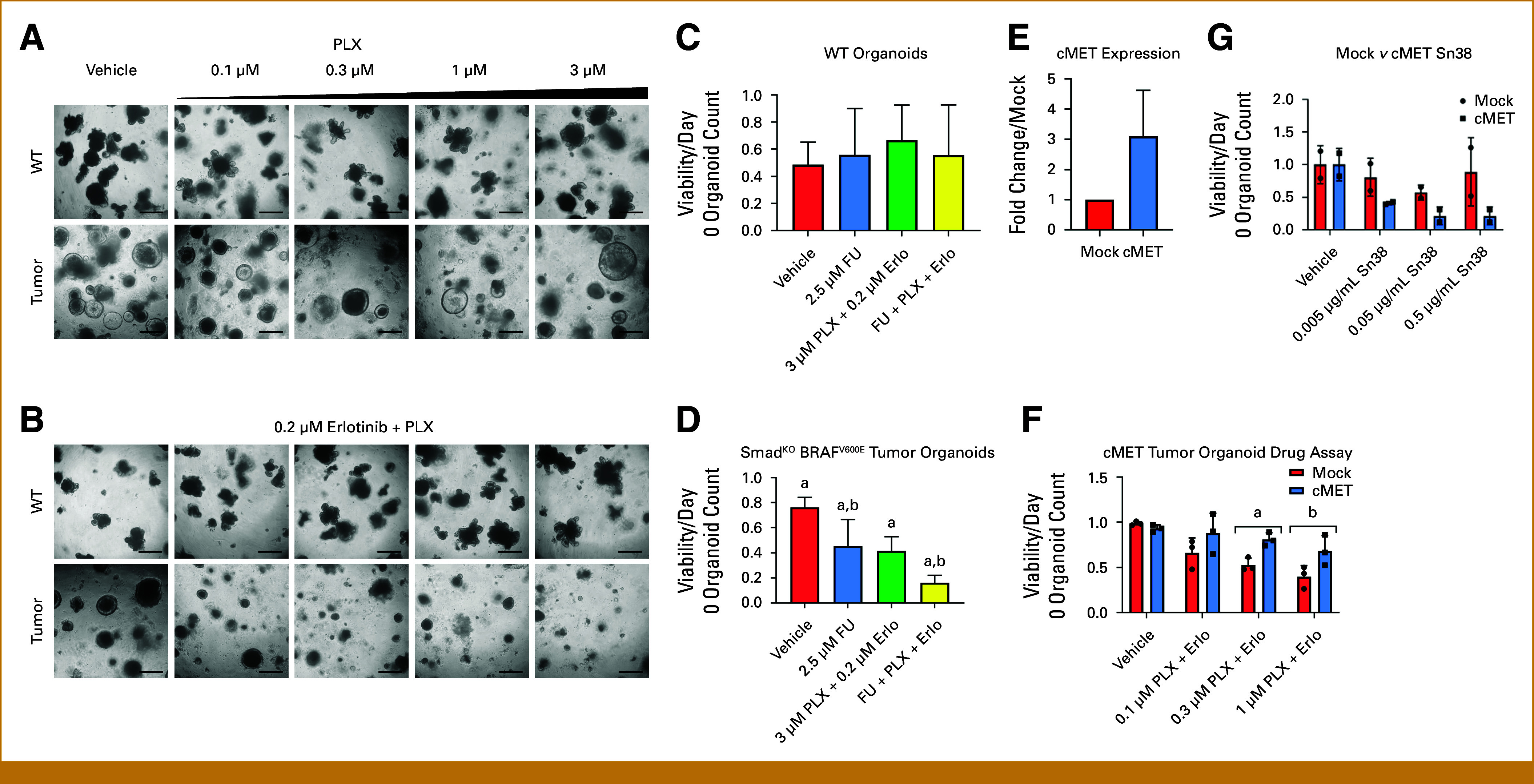
Modeling response to chemotherapy and targeted therapy in Smad4^f/f^; Braf^V600E/+^ organoids. (A) Wildtype and *Smad4*^*f/f*^; *Braf*^*V600E/+*^ tumor organoids were cultured for 3 days and then treated with vehicle, or indicated concentrations of vemurafenib (PLX) alone, and (B) 0.2 µM erlotinib alone, or indicated concentrations of PLX supplemented with 0.2 µM erlotinib for 14 days. Representative images of three biologic replicates (scale bar = 100 µm). Although wildtype organoids are unaffected by these treatment regimens, tumor organoids were susceptible to combination therapy. (C, D) Additive effect of treating *Smad*^*f/f*^; *Braf*^*V600E/+*^ tumor organoids with FU and targeted therapy. (C) WT or (D) tumor organoids were treated with regular culture media for 3 days and then treated with vehicle, 2.5 μM FU, 3 μM PLX + 0.2 μM erlotinib, or a combination of all 3 (3 μM PLX + 0.2 μM erlotinib + 2.5 μM FU) for 7 days. Viability counts were determined and standard deviations of three biologic replicates are indicated by error bars (a = *P* < .05 *v* vehicle; b = *P* < .05 *v* 2.5 μM FU alone, ANOVA). (E) qPCR of cMET expression in *Smad*^*f/f*^; *Braf*^*V600E/+*^ tumor organoids. (F) Mock and lentiviral-transduced cMET-expressing Smad4^f/f^; Braf^V600E/+^ tumor organoids were cultured as described above treated with vehicle or varying concentrations of PLX supplemented with 0.2 μM erlotinib for 7 days. Viability counts for organoids treated with PLX + erlotinib were determined and standard deviations of three biologic replicates are indicated by error bars (a = *P* < .05; b = *P* = .076). Inset shows plot of RT-PCR measurement of MET RNA in vector-transfected and MET-transfected organoids. (G) Mock and lentiviral-transduced cMET-expressing Smad4^f/f^; Braf^V600E/+^ tumor organoids were treated with increasing doses of SN38 and viability counts. Standard deviation of two biologic replicates are shown. ANOVA, analysis of variance; FU, fluorouracil; PLX, vemurafenib (PLX4032); qPCR, quantitative polymerase chain reaction, RT-PCR, reverse transcriptase polymerase chain reaction; WT, Wilms tumor.

To model the response of BRAF-V600E-mutant CRC to both targeted therapy and chemotherapy, we used a *Smad4*^*f/f*^; *Braf*^*V600E/+*^ mouse model, which emulated the patient's mutational profile.^10-12^ Organoids were prepared from wildtype intestinal crypts and from tumors found in *Smad4*^*f/f*^; *Braf*^*V600E/+*^ mice (n = 3). To assess whether the tumor organoids mimic drug sensitivity found in patients, organoids were treated with the BRAF inhibitor vemurafenib (PLX4032, PLX), the EGFR inhibitor, erlotinib, or a combination of both. Wildtype organoids did not show sensitivity to any of the treatments, indicating that the concentrations of inhibitors are not cytotoxic to normal intestinal epithelium (Figs 4A and 4B). Supporting previous findings that BRAF-V600E colon tumors are resistant to treatment with vemurafenib,^19^ *Smad4*^*f/f*^; *Braf*^*V600E/+*^ organoids were not sensitive to vemurafenib alone, or to treatment with erlotinib (Figs 4A and 4B). By contrast, the tumor organoids did show sensitivity to the combination of erlotinib and vemurafenib even at lower concentrations of the BRAF inhibitor (Fig 4B). These data demonstrate that our BRAF-V600E organoid model recapitulates the resistance and sensitivity to inhibitor treatments seen in human patients with BRAF-V600E-mutant colon cancer.

**FIG A1.**
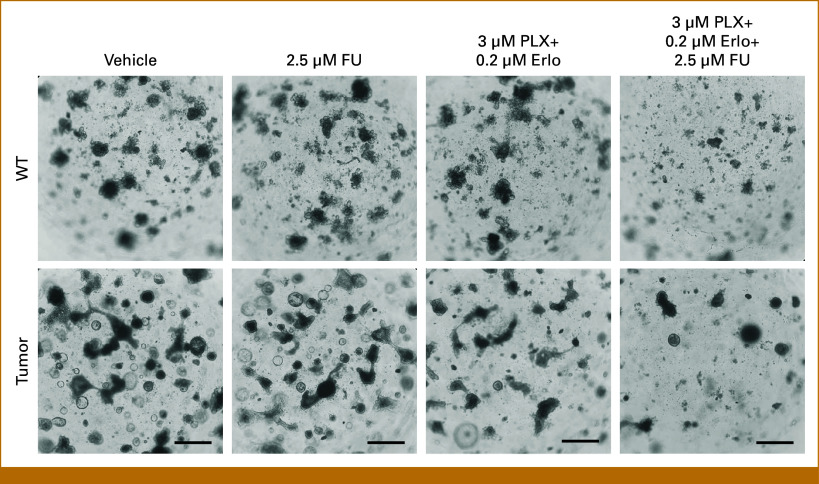
Images of organoid sensitivity to combinatorial treatment. Passaged organoids were treated with regular culture media for 2 days and then treated with vehicle, 2.5 µM FU, 3 µM PLX + 0.2 µM Erlo, or a combination of all 3 for 7 days. Images are representative of three biologic replicates. Viability counts were determined by observing the deterioration of healthy organoid structural border and appearance of debris. Standard deviation of three biologic replicates are indicated by error bars. Scale bar = 0.5 mm. Erlo, erlotinib; FU, fluorouracil; PLX, vemurafenib (PLX4032); WT, Wilms tumor.

The effect of combining FU with EGFR and BRAF inhibition was evaluated, as these treatments appeared to be non–cross-resistant in the patient data. As above, wildtype and *Smad4*^*f/f*^; *Braf*^*V600E/+*^ tumor organoids were treated with FU, PLX + erlotinib, or a combination of all three drugs. In all treatment conditions, wildtype organoids were largely unaffected by any treatment regimen. By contrast, *Smad4*^*f/f*^; *Braf*^*V600E/+*^ tumor organoids showed significant decreases in viability in all combinations tested when compared with vehicle treatment (Figs 4C and 4D, Appendix Fig A1). Strikingly, the combination of EGFR/BRAF inhibition with FU was significantly more effective than either treatment alone in this organoid model.

**FIG A2.**
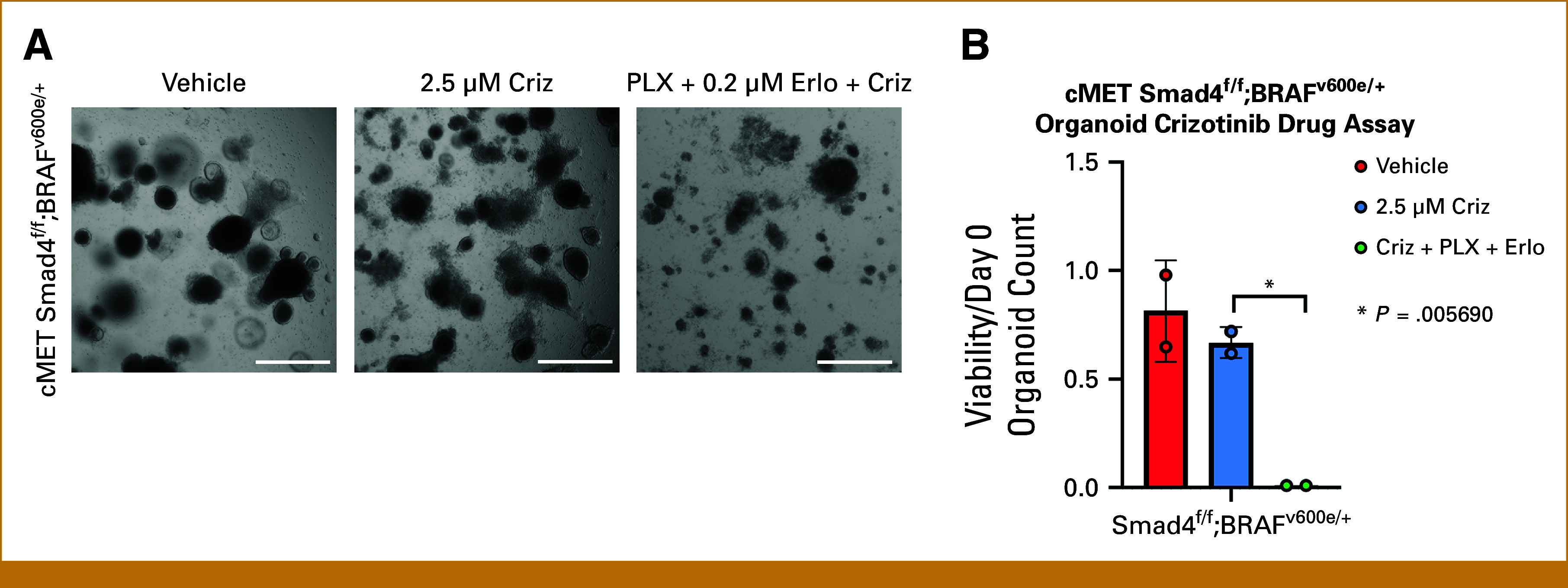
Criz induces death in cMET organoids when combined with PLX and Erlo. (A) Representative 4× images of biologic replicates of cMET Smad4^KO^Braf^V600E/+^ (N = 2) organoids treated with vehicle or 2.5 μm Criz + 0.3 μm PLX + 0.2 μm Erlo. Scale bar = 0.5 mm. (B) cMET organoids were treated with vehicle, 2.5 μM Criz, or 2.5 μm Criz + 0.3 μm PLX + 0.2 μm Erlo, and viability was assessed 7 days after treatment. Criz, crizotinib; Erlo, erlotinib; PLX, vemurafenib (PLX4032).

The human patient case study revealed a population of MET-amplified tumor cells that were resistant to EGFR-/BRAF-targeted therapy. To investigate the role of MET amplification in mediating resistance to EGFR-/BRAF-targeted therapy, cMET expression was induced by stable transgene expression in *Smad4*^*f/f*^; *Braf*^*V600E/+*^ organoids (Fig 4E). Mock-infected and cMET-expressing tumor organoids were treated with a combination of erlotinib and PLX as above. Although mock-infected organoids show a drop in viability when treated with the combination of erlotinib and PLX, negatively correlating with increasing concentration of PLX, tumor organoids expressing cMET were resistant to this combined treatment regimen (Fig 4F), but regained sensitivity when MET-inhibitor crizotinib was added^20^ (Appendix Fig A2). Thus, the organoid model system supports the notion that amplification of MET confers resistance to BRAF-/EGFR-targeted therapy in BRAF-mutant CRC, as seen in our patient.

**FIG A3.**
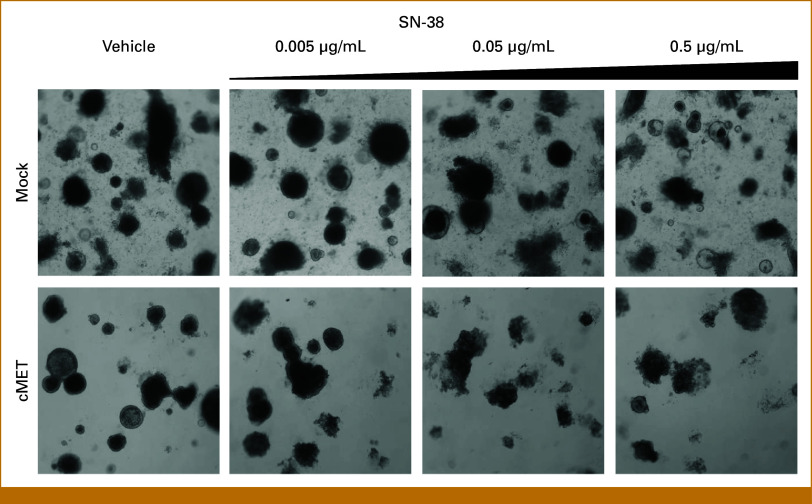
Images of cMET organoid sensitivity to SN38 treatment. Representative images of Smad4^KO^Braf^V600E/+^ tumor organoids (mock or cMET lentiviral transfected, two biologic replicates) treated with increasing concentrations of SN38 for 7 days.

The patient's tumor developed resistance to BRAF/MEK/EGFR inhibition with MET amplification, but remained sensitive to FOLFIRI, suggesting MET amplification did not induce resistance to irinotecan. Mock-infected and MET-expressing *Smad4*^*f/f*^; *Braf*^*V600E/+*^ organoids were treated with increasing doses of SN38, the active metabolite of irinotecan. Expectedly, cMET-expressing tumor organoids were not resistant to SN38 treatment when compared with the parental organoids, and may be somewhat more sensitive (Fig 4G, Appendix Fig A3). These data demonstrate that MET amplification does not induce resistance to chemotherapeutic agents.

## DISCUSSION

BRAF-V600E-mutant CRC have a poor prognosis and are resistant to BRAF and MEK inhibitor treatment because of adaptive activation of EGFR signaling. Combined treatment with EGFR and BRAF inhibitors with or without MEK inhibitors overcomes this adaptive mechanism and can lead to clinically significant responses.^18^ However, the responses often are not durable and patients soon develop resistance and disease progression.^17^

The pediatric patient discussed in this study presented with metastatic, MSS, BRAF-V600E colorectal adenocarcinoma. She was treated with alternating chemotherapeutic and targeted therapy regimens on the basis of tumor evolution seen in serial plasma sequencing at regular intervals throughout the course of treatment. These alterations are reflective of underlying tumor cell clonal evolution with selection for clones harboring resistance mechanisms in the presence of initially effective treatment. The responses to both chemotherapy and targeted therapy were not durable and this may be due to the known aggressiveness of MSI-stable BRAF-mutant colon cancers, especially those that, like in this patient, do not harbor RNF43 mutations.^21^ Very high BRAF variant allele frequencies in plasma were also noted at progression, which is also associated with aggressive disease.^22^

Initial resistance to BRAF/MEK and EGFR inhibition was associated with development of both BRAF and MET amplification, seen in both cfDNA assays and genomic analysis of liver metastases. MET amplification has been shown to be a mechanism of acquired resistance to combined BRAF and EGFR therapy in BRAF-V600E-mutant CRC and can respond to crizotinib.^9^ However, in this case, the patient was treated and responded to second-line chemotherapy FOLFIRI. On FOLFIRI, the previously shown BRAF or MET amplification did not reappear, suggesting that the BRAF-MET-amplified clones that rendered resistance to PDT therapy did not expand or were eliminated during FOLFIRI treatment. Of note, the sensitivity of cell-free DNA assays at identifying and quantitating copy-number alterations can be low if there is low amount of tumor DNA present; in our case, the high VAFs of BRAF present suggest that lack of identification of MET amplification was not a false negative. Additionally, MET expression in BRAF-V600E tumor organoids induced resistance to BRAF/EGFR inhibition but remained chemosensitive to SN38, suggesting that the mechanism of resistance to targeted therapy for BRAF-V600E does not induce resistance to chemotherapy.^23^ Similar findings have been reported in colon cancer, where KRAS-mutant clones selected during resistance to EGFR antibody therapy regressed when EGFR antibody was discontinued,^24^ and in melanoma, where clones harboring BRAF fusions selected during development of resistance to vemurafenib regressed when targeted therapy was discontinued.^25^

**FIG 5.**
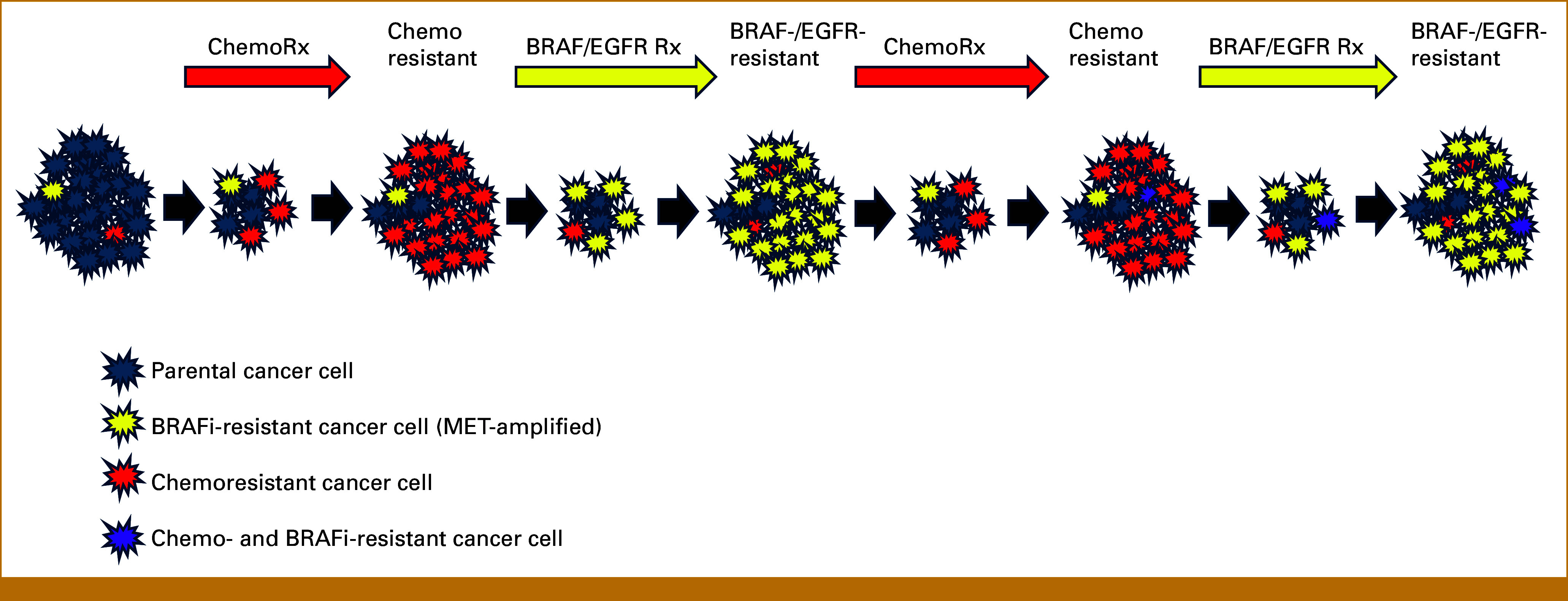
Illustration of model of clonal dynamics. Before treatment, there may be preexisting clones that may be resistant to chemotherapy or targeted therapy, but these do not have fitness advantage over parental tumor cells in the absence of treatment. Selection pressure from effective chemotherapy led to selection of chemoresistant clones, but these remained sensitive to therapy targeting BRAF/EGFR. Treatment targeting BRAF/MEK/EGFR in these chemoresistant tumors led to response and eventual selection of a different clone of BRAF-/EGFR-resistant clones (harboring MET amplification) that led to progression. These clonal dynamics are shown over multiple cycles, with possible selection for panresistant clones with time.

These observations support several important findings. (1) Targeted therapy aimed at BRAF/MEK and EGFR is non–cross-resistant to standard chemotherapy; and (2) resistance to therapy is not necessarily cumulative and irreversible, but instead can be transient and driven by rapid clonal dynamics under differing selection pressures (Fig 5). The pattern of response in different liver metastases varied widely, demonstrating spatial heterogeneity in composition of resistant clones in different regional metastases. This latter observation suggests a rationale for considering locoregional treatment to eliminate specific resistant metastases in some settings of oligoprogression in colon cancer therapy. This approach will require careful consideration of potential toxicity of locoregional management and management of systemic therapy.

Our findings strongly suggest that standard chemotherapy and targeted therapy inhibiting BRAF/MEK and EGFR impose quite different selection pressures on the tumor cells (Fig 5). This is consistent with other reports showing efficacy of rechallenge with BRAF-/EGFR-targeted therapy.^26^ Therefore, combining targeted therapy and chemotherapy, or upfront rapidly alternating therapy with BRAF/MEK and chemotherapy may be able to exert a dual selection pressure and delay development of resistance. Our findings also lend support to future and ongoing studies that use serial cfDNA profiling to monitor clonal evolution and inform change of therapy, including rational rechallenge with previous regimens.

Finally, our findings highlight the efficacy of using organoid models to recapitulate patient tumor drug sensitivities, revealing the potential of using organoids as a translational model.^27-29^ Tumor organoids obtained from a BRAF-V600E-driven mouse model of intestinal cancer demonstrated that combined treatment with BRAF and EGFR inhibition and FU is more effective than either modality alone, supporting a role for combined targeted and chemotherapy for MSS-stable, BRAF-V600E-mutant CRC. BRAF-V600E-mutant cancers with underlying mismatch repair deficiency are likely to be resistant to FU-based therapy and may require different combination treatment strategies, possibly with addition of immunotherapy.^25^ The establishment of a model to study immunotherapies in an immune-organoid coculture system is an exciting field to pursue.^30,31^ Thus, organoid models can be a powerful tool to investigate combinations and sequential treatment regimens aimed at maximizing response and minimizing chance of acquired resistance.

## Data Availability

Available upon request.

## APPENDIX

**TABLE A1.** Mutations and Mutant Allele Frequencies Identified in Serial Circulating Tumor DNA Analyses

Day	BRAF V600E	TP53 G245S	SMAD S405R	PIK3CA E453D	CCNE1 A353D	ERBB2 C584G	ARID1A R693R
148	0.2	0.2	0.2	0	0	0	0
191	0	0	0	0	0	0	0
253	25.6	5.4	4.1	0	0	0	0
291	4.9	1.3	0.7	0	0	0	0
337	0	0	0	0	0.1	0.4	0.5
372	0.2	0	0	0	0	0	0
411	1.2	0.8	0.7	0	0	0	0
442	26.2	19.2	20.7	0.4	0	0	0
467	0.3	0.1	0	0	0	0	0
519	0.4	0.1	0	0	0	0	0
571	9.3	2.5	2.1	0	0	0	0
632	42.3	12.8	8.9	0	0	0	0
